# Hemoptysis—Report of Three Cases Treated by Artificial Pneumo-Thorax

**Published:** 1916-01

**Authors:** Alvis E. Greer

**Affiliations:** Houston, Texas


### TEXAS MEDICAL JOURNAL

**Dr. F. E. Daniel, Founder. Established July, 1885**

MRS. F. E. DANIEL, - Publisher and Managing Editor

Published Monthly.—Subscription, $1.00 a Year.

VOL. XXXI AUSTIN, JANUARY, 1916 No. 7

The publisher is not responsible for views of contributors

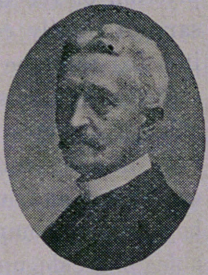

### Original Articles.

### Hemoptysis—Report of Three Cases Treated by Artificial Pneumothorax.

BY ALVIS E. GREER, M. D., HOUSTON, TEXAS.

The proper treatment of pulmonary hemorrhage in tuberculosis has been a subject of much thought and experimentation. Many drugs have been used to stop the bleeding by their action upon the blood pressure, blood coagulability, respiratory excursion and cough, combined with rest, ice-bags to the chest, etc. Their multiplicity may explain their lack of individual value. With the introduction of lung compression by nitrogen gas in these cases a step forward was taken.

When we pause to consider the pathology of most of these cases of pulmonary hemorrhage we are the more impressed with the rationale of the method. A tuberculous infiltration breaking down may ulcerate into a blood-vessel of variable size; the hemorrhage will be large or small according to the size of the vessel eroded. It is true that tuberculous patients as a rule do not die of these hemorrhages per se. But repeated insults upon their blood supply will the more exhaust already failing strength. We would, perhaps, despair of stopping the bleeding from an artery of the leg if the patient were allowed to run and our method of treatment consisted of nitroglycerin, calcium, normal salt solution, etc. Picture a cavity in the lung with a bare artery pumping with every heart-beat and the air rushing by its walls with every respiratory phase; an ice-bag on the chest probably three inches from the bleeder; normal salt being injected into the vein; calcium and magnesium sulphate by mouth. A rational method!

There are certain of these cases which can be treated by compression of the lung by nitrogen. In some cases it is not necessary to tightly compress the whole lung; 400 or 500 c.c. of gas injected into the pleural cavity near the bleeding area may exert sufficient compression to stop the bleeding. The cases most suitable for compression by nitrogen gas are the early cases of hemoptysis in lung tuberculosis. As a general rule, pleural adhesions are absent and, when present, they are limited and the lung can be partially compressed. It is of course necessary for one to be able to decide upon the side from which the bleeding is coming. But in these cases it is usually easy to decide. Even in bilaterally involved cases the onset of new bubbling rales in an area of diminished breath sounds in one lung due to its having been flooded with blood is good evidence of the side affected.

Dense pleural adhesions will cause a failure in some cases. A very active focus on the opposite side may contraindicate compression of the bleeding lung. Complicating cardiac diseases, especially of a myocardial character, constitutes a condition, the existence of which should cause one to well consider the advisability of throwing a whole lung out of commission.

The advantages of immediate compression of the lung by gas are:

1. Immediate and permanent cessation of bleeding and the saving of a great deal of blood to the patient.

2. Less likelihood of a secondary broncho-pneumonia, which at times occurs in the blood-soaked lung treated without compression.

3. Lessened period of confinement to bed and a great deal less discomfort and suffering to the patient.

4. The compression can be maintained with its added beneficial influence upon the arrest of the tuberculous process.

Up to the present time we have had three cases of frank pulmonary hemorrhage treated by gas compression. One was a case of hemoptysis occurring as the initial symptom of tuberculosis; the other two cases were in moderately advanced tuberculous individuals. Other measures to control the bleeding were faithfully tried on all three patients but without result. The histories follow:

Case No. 1.—J. R. K., male; dentist's assistant; white; single; age, 20; duration of disease, one and one-half years; mother and brother died of tuberculosis; usual weight, 125 pounds; present weight, 100 pounds.

This boy had been under my observation for some time, and he was steadily and rapidly becoming worse. His afternoon temperature was 101 to 103 F. He had several hemorrhages of slight amounts. His sputum was purulent and expectoration free, eight ounces in twenty-four hours, and at times blood-tinged. Night sweats were annoying, and he was very weak. Physical examination showed that the right upper and middle lobes were considerably involved with a moderately large cavity in the upper lobe. The left upper lobe was also somewhat involved. These findings were corroborated by an X-ray examination by Dr. Edgar H. Lancaster. He developed a small hemorrhage which did not cease, and he continued to expectorate a small amount of blood each day. On June 17, 1914, I injected 400 c.c. of nitrogen gas into the right pueral cavity. Following this injection his hemorrhage ceased and for the next two days his afternoon temperature was 101. It then fell gradually as follows (maximum afternoon readings): 22d, 100; 23d, 100; 24th, 99½; 25th, 98.6. Because of this marked improvement, I decided to completely collapse the lung. A hemorrhage did not recur, and the patient improved rapidly up until the time I last saw him, October 14, 1914. At this time he was expectorating about one-eighth cup of sputum during each twenty-four hours. His temperature was normal and he looked greatly improved. A letter dated June 25, 1915, states that he is feeling well and that he thinks his disease is arrested.

Case No. 2.—A. (seen through the courtesy of Dr. Louis Daily); Russian Hebrew; age, 23; shoemaker; left-sided tuberculosis, most marked in the upper lobe. Profuse hemorrhages had been in evidence for two weeks. All usual methods had failed to stop the bleeding. His maximum afternoon temperature had been 102. He gave a history of having had some tuberculous trouble for three years. The left side of the chest was full of mucous rales; there were none on the right side. The left lung was collapsed with oxygen gas and, because of the rapid absorption of the oxygen, injections were given every other day. Following the first injection there were no hemorrhages for eighteen hours, when a hemorrhage occurred following an enema; patient became very excited before this hemorrhage. Thirty-six hours passed without a hemorrhage, when after the excitement and exertion of an enema a hemorrhage of small amount took place, followed by two slight hemorrhages in the next six hours and a more considerable one eight hours afterwards. The next day I injected 1000 c.c. of oxygen gas attaining an intrathoracic pressure of positive 10½ cm. water. The patient suffered no inconvenience during or following the injection. There were no hemorrhages until the second day, when a hemorrhage of four ounces took place. Since the hemorrhages had been taking place on the second day after the injection, I decided that it was due to the fact that the oxygen gas, which we were forced to use because of lack of nitrogen gas, was being absorbed so rapidly that the intra-pleural pressure decreased to such an extent that it allowed the bleeding to continue. I therefore injected the oxygen daily, and following these injections there were no further hemorrhages.

CASE No. 3.—R. F., aged 35; American; married; solicitor (seen through the courtesy of Dr. M. B. Stokes). Felt perfectly well until the evening of June 26, 1915. While lying in bed reading he had a pulmonary hemorrhage. The total amount of bleeding that night was about one-half tea cup. He bled a small amount every day or two for the next week. During this time every effort was made to control the bleeding but without avail. Tubercle bacilli were found in the sputum. Physical signs found were bubbling rales at the junction of the upper right and middle lobes of the right lung. It was decided to compress the lung with nitrogen gas, and 900 c.c. were injected into the right pleural cavity. Four hours after this injection the patient, against my orders, walked to the bath room and on his way back to bed fell over a chair. A slight amount of bleeding took place at this time. The next day 1000 c.c. of nitrogen were injected into the right pleural cavity and following this injection there were no further hemorrhages. Four days later the patient was sent to San Angelo, Texas, where he is at the present time and doing well, and he has had no further hemorrhages.

From a consideration of the results obtained in these cases, we feel that we are justified in concluding:

1. That compression of the affected lung in the hemoptysis of tuberculosis offers the safest, quickest and most rational method of controlling the hemorrhage. The compression should be continued until fibrosis takes place in the cavity.

2. In moderately advanced cases the lessening of the toxemia is marked.

3. The opposite "good" lung stands the extra work well. It is of course necessary that any infection in the good lung be in a fairly inactive state. In cases Nos. 1 and 2 the opposite lung was involved.

4. Oxygen is not as suitable a gas to use as is nitrogen because oxygen is so rapidly absorbed. However, the general condition of the patient—case No. 2—seemed to be better after an injection than could be reasonably accounted for by cessation of the bleeding. It has occurred to us that it would be of benefit to compress the lung by nitrogen gas and at the same time inject oxygen gas subcutaneously.

